# Microbial diversity arising from thermodynamic constraints

**DOI:** 10.1038/ismej.2016.49

**Published:** 2016-04-01

**Authors:** Tobias Großkopf, Orkun S Soyer

**Affiliations:** 1 School of Life Sciences, University of Warwick, Coventry, UK

## Abstract

The microbial world displays an immense taxonomic diversity. This diversity is manifested also in a multitude of metabolic pathways that can utilise different substrates and produce different products. Here, we propose that these observations directly link to thermodynamic constraints that inherently arise from the metabolic basis of microbial growth. We show that thermodynamic constraints can enable coexistence of microbes that utilise the same substrate but produce different end products. We find that this thermodynamics-driven emergence of diversity is most relevant for metabolic conversions with low free energy as seen for example under anaerobic conditions, where population dynamics is governed by thermodynamic effects rather than kinetic factors such as substrate uptake rates. These findings provide a general understanding of the microbial diversity based on the first principles of thermodynamics. As such they provide a thermodynamics-based framework for explaining the observed microbial diversity in different natural and synthetic environments.

## Introduction

There is an immense diversity of microbes in the natural environment (Curtis *et al.*, 2002). One major challenge for microbial ecology, besides achieving more complete enumeration of the total diversity (Bunge *et al.*, 2013), is to explain how this diversity is generated and maintained over evolutionary time. In particular, understanding the set of environmental, biochemical and evolutionary conditions that can lead to the generation and maintenance of microbial diversity is a prerequisite to understand and control natural microbial populations (Gudelj *et al.*, 2010; O'Brien *et al.*, 2013) and engineer synthetic microbial communities (Großkopf and Soyer, 2014).

In ecology, a historically dominant idea in the study of diversity is the ‘competitive exclusion principle', which states that at equilibrium no two species can coexist occupying the same niche (Hardin, 1960). This principle is shown theoretically in the context of microbial ecology using mathematical models of the well-mixed, single substrate chemostat environment. In such environments, the theory predicts that coexistence of two species can only be possible for a unique combination of kinetic parameters, and outside this combination only a single species, that has the highest substrate affinity, can survive at steady state (Hsu *et al.*, 1977). Thus, it is expected that a single organism should monopolise each substrate; and the number of observed species in the environment should not surpass the number of limiting nutrients or substrates. This exclusion theory led to the proposition of the ‘paradox of the plankton' as the problem of how a high biological diversity can be maintained on a relatively limited number of niches (Hutchins, 1961). One way to resolve this paradox is to invoke spatial and temporal variations in substrate levels (Hsu *et al.*, 1977; Pfeiffer *et al.*, 2001; Kerr *et al.*, 2002; MacLean and Gudelj, 2006).

Although spatial and temporal variation in a single substrate can certainly contribute to microbial diversity, it cannot explain species harbouring different metabolic pathways that enable conversion of the same substrate into different end products (Rodríguez *et al.*, 2008). Even a common substrate such as glucose can be converted into a variety of end products by different species or even within a single species (Gupta and Clark, 1989). This catabolic diversity contributes to the observed species diversity in the environment; and it is possible that these two observations of diversity are linked. For example, it is proposed that metabolic byproducts (Schink, 1997) as well as specific production of toxic substances (Lenski and Hattingh, 1986) could differentially inhibit competing species or result in autoinhibition (De Freitas and Fredrickson, 1978). When such inhibition affects species competing for a given substrate in a differential way, it can allow coexistence on that single substrate (De Freitas and Fredrickson, 1978; Lenski and Hattingh, 1986).

Arguably the most fundamental inhibitory constraint on microbial growth is that arising from thermodynamics of metabolism. As microbial growth depends on energy harvested from substrates converted into end products, it is governed by the thermodynamics of such metabolic conversions. Here, we consider this relation between the thermodynamic constraints placed on growth sustaining metabolic conversions and the resulting population-level dynamics. Using thermodynamic models of microbial growth, we show that the inevitable slowing down of microbial growth ensuing from product built-up can lead to coexistence of different species implementing different metabolic conversions and consuming the same substrate. As each metabolic conversion operates with different thermodynamics, species utilising these are governed by different growth and product-inhibition dynamics that result in their coexistence. We find this ‘thermodynamics inhibition' effect to be strongest for reactions leading to a low change in free energy, where it dominates over kinetic factors such as substrate uptake rates. In line with this fundamental observation, we find that several biologically relevant microbial conversions as well as theoretically possible metabolic conversions of glucose fit in the regime of strong thermodynamic effects and readily lead to coexistence of different species on a single substrate. These findings provide a thermodynamic basis to evaluate observed microbial diversity.

## Materials and methods

### Thermodynamic model for microbial growth

The change of free energy generated by a metabolic conversion under non-standard conditions can be calculated according to Equation 1;


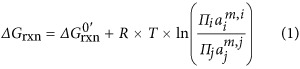


where *R* is the gas constant, *T* is the temperature, a and *m* are the chemical activity and stoichiometric coefficient of each compound involved in the reaction, *i* and *j* are indices over products and substrates, respectively, and 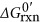 is the change in free energy under biological standard conditions (1 m concentration of all solutes, 1 atm, 25 °C, and pH 7).

We use here a previously proposed thermodynamic model (Hoh and Cord-Ruwisch, 1996) that captures the observed microbial growth dynamics under anaerobic, low-energy conditions. Utilising the results from reversible enzyme kinetics (Haldane, 1930), this model derives a rate function for microbial growth as follows;


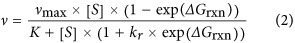


Here 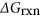 is the thermodynamic energy available in the reversible reaction for a given set of substrate and product concentrations, as given in its generic form in Equation 1. The constants *K* and *k*_*r*_ are the half saturation constant for substrate turnover and the ratio of maximum forward over maximum reverse reaction rate, respectively (Hoh and Cord-Ruwisch, 1996).

Note that by modelling microbial growth as a reversible enzymatic reaction, this model makes the assumption that the rate of such an equation can be taken as the growth rate. In reality, the available thermodynamic energy in the reaction would have to be invested in driving the reaction, as well as in growth and cellular maintenance. Subsequent studies have tried to address this point by proposing alternative models that consider the connection between the energetics of catabolic and anabolic metabolism (Kleerebezem and Stams, 2000; Jin and Bethke, 2003, 2007; Rodríguez *et al.*, 2008). In the context of the present study, any of these more complex models could be utilised without affecting the key conclusion that thermodynamic inhibition can lead to microbial diversity on a single substrate (see Supplementary Text). Implementing these models, we find that conclusions regarding the interplay of kinetic and thermodynamic factors counteracting each other (Figure 5) are affected only quantitatively by the exact model choice on how much of the free energy available from a reaction is invested in growth rate vs biomass production. The presented results provide a conservative estimate.

### Kinetic, non-thermodynamic model for microbial growth

For comparison with the thermodynamic model, we also use a solely kinetic model first developed by Monod (1949). This model uses an empirically derived kinetic equation to describe microbial growth and does not include the effects arising from thermodynamics. The reaction rate for microbial metabolism is given by


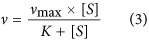


Note that this equation results also from the thermodynamic model (Equation 2) when the change in free energy available from the growth reaction is very negative (that is, exp(*ΔG*_rxn_)≈0).

### Modelling growth of two species in a chemostat on the same substrate

We consider two species *X*_1_ and *X*_2_ in a chemostat, where they are consuming the same substrate, *S*. Species *X*_1_ and *X*_2_ are assumed to process the substrate to produce different end products, *P*_1_ and *P*_2_, respectively. The feed-in rates of the species to the chemostat are considered to be zero, while the substrate concentration in the feed is given by *S*_0_. The dilution rate (per hour) of the chemostat is considered to be *λ*. Using these notations, and a given microbial growth model, we can construct ordinary differential equations (ODEs) to model the dynamics in a chemostat as follows:


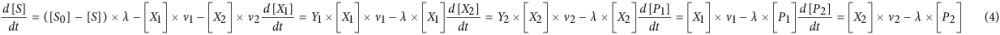


where *Y* is a biomass yield parameter and *v*_*i*_ represents the reaction rates of species *i* as given by Equations 2 or 3. To understand the species concentrations at steady state, we can solve Equation 4 by setting the left side of each of the ODEs equal to zero. In particular, we can use the steady-state condition for second and third ODEs to obtain:


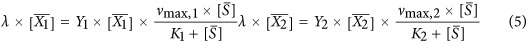


where the bar notation indicates steady-state concentrations. For the non-thermodynamic model, this condition can only be satisfied if *ν*_1_ × *Y*_1_=*ν*_2_ × *Y*_2_, which can be achieved only by a unique combination of the yield, maximal growth and uptake parameters or when all these parameters are the same (that is, *X*_1_ and *X*_2_ are the same species) (Hsu *et al.*, 1977). Under any other set of parameters, the species with the lower affinity for the substrate will be washed out of the chemostat (Hsu *et al.*, 1977).

For the thermodynamic model, the steady-state condition leads to


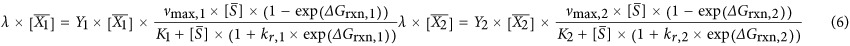


Assuming that *k*_*r1,2*_=1 (equal maximum forward and backward conversion rates) and parameters *K*_1_ and *K*_2_ are much larger compared with the steady-state substrate concentration, we can re-arrange this equation to derive a condition for the steady state as


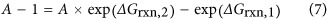


where A is a composite parameter given by 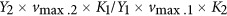. Thus, steady-state condition can be satisfied with the two species coexisting, for the correct combination of their kinetic parameters and metabolic free energy changes. Since metabolic free energies are a function of substrate and product concentrations, coexistence is possible in a larger dynamical regime compared with the kinetics-only model. To get a sense on how species frequencies at steady state depend on product concentrations, we can make the strict assumption that yield, maximal growth and uptake parameters of the two species are the same (this could be the case right after a speciation event), that is, *A*=1. In this case, we can substitute Equation 1 into the simplified Equation 7 to derive a relation between the end product concentrations at steady state as a function of the standard free energy changes of the metabolic conversions:


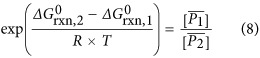


Since the product concentrations at steady state relate to species concentrations at steady state (see Equation 4 and Supplementary Text), the relation given by Equation 8 extends to the species concentrations. We conclude that the two species with equal kinetic parameters will coexist at steady state at frequencies that are determined by the standard free energy changes of the metabolic conversions that they utilise as given in Equation 8.

### Sampling of metabolic reactions from glucose

To analyse the prevalence of reactions with low Gibbs free energy change, we consider stoichiometrically balanced, fermentation reactions starting from glucose and involving 12 additional common compounds. We generate all possible reactions among these compounds, where each reaction must feature glucose as a substrate. To automate this reaction generation, we define stoichiometric coefficient ranges for each compound, where negative and positive coefficients indicate compounds taking part in a reaction as product or substrate, respectively. We then computationally iterate through these stoichiometric coefficient ranges to generate all possible reactions within these limits. The 13 compounds used in this analysis and their stoichiometric ranges are (lower bound; upper bound): glucose (−1;−1), lactate (0;2), acetate (0;3), CO_2_ (−6;6), formate (0;3), pyruvate (0;2), acetaldehyde (0;2), ethanol (0;2), H_2_ (0;6), H^+^ (0;12), water (−6;6), butyrate (0;2) and methane (0;6).

As an example, the homolactic fermentation of Glucose would be represented by the following vector of stoichiometric coefficients for these compounds: [−1,2,0,0,0,0,0,0,0,2,0,0,0]. All reaction vectors within the search space are generated (that is, all combinatory permutations) and are then evaluated for chemical and stoichiometric balance. To do so, we multiply each reaction vector with a mass- and charge-balance matrix that holds the number of carbon, hydrogen and oxygen atoms as well as the charge of each molecule (see Supplementary Table S1). For a reaction to be balanced, this operation must yield zero. Balanced reactions are then considered as biochemically feasible and their Gibbs free energy change is computed, assuming biological standard conditions (1 m, 1 atm, 298.13 °K, pH=7) and using tabulated formation energies (Thauer *et al.*, 1977). The full list of reactions resulting from this analysis is given in Supplementary Table S2. A MATLAB script that implements the above algorithm is provided in Supplementary File and can also be found at http://osslab.lifesci.warwick.ac.uk/?pid=resources.

## Results

To analyse the relation between cellular metabolism and microbial ecology, we consider the thermodynamic basis of microbial growth. Microbes achieve cellular growth through harvesting of free energy change available from metabolic conversions. As with any other chemical reaction, these conversions are governed by thermodynamics whereby the free energy change for the reaction is given by the difference between the free energy of formation of the products 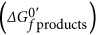 and the substrates 
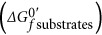
 in that reaction. This is the maximum theoretical amount of energy that microbes can utilise from the degradation of a substrate for building up their own cell biomass under standard biochemical conditions. If the concentrations deviate from standard conditions (1 m concentration of all solutes, 1 atm, 25 °C, and pH 7), the apparent thermodynamic energy Δ*G*_rxn_ available for microbial growth can be calculated according to Equation 1. In reality, energy available for growth would be less than this maximum amount, due to energy investments in driving anabolic reactions leading to biomass and other maintenance requirements (Thauer *et al.*, 1977; Hoh and Cord-Ruwisch, 1996; Jin and Bethke, 2007; Rodríguez *et al.*, 2008) (see further discussion below).

### Thermodynamic constraints allow for coexistence of multiple species on a single substrate

To account for the inherent thermodynamic constraints on microbial metabolism given by Equation 1, we use here a thermodynamic growth model (Hoh and Cord-Ruwisch, 1996) (see Materials and methods). This model is shown to provide a better explanation of microbial growth under anaerobic, low-energy conditions, where thermodynamic effects are expected to be more profound, compared with models that are solely based on empirical, kinetic formalisms (Hoh and Cord-Ruwisch, 1996). Within this model, the growth rate of each species is described by a function that contains kinetic and thermodynamic factors that are governed by the substrate uptake dynamics and the free energy of the reaction converting substrates to products respectively (see Equation 2). Considering alternative thermodynamic models does not alter qualitative conclusions of the presented study (see Materials and methods and Supplementary Text).

**Figure 1:**
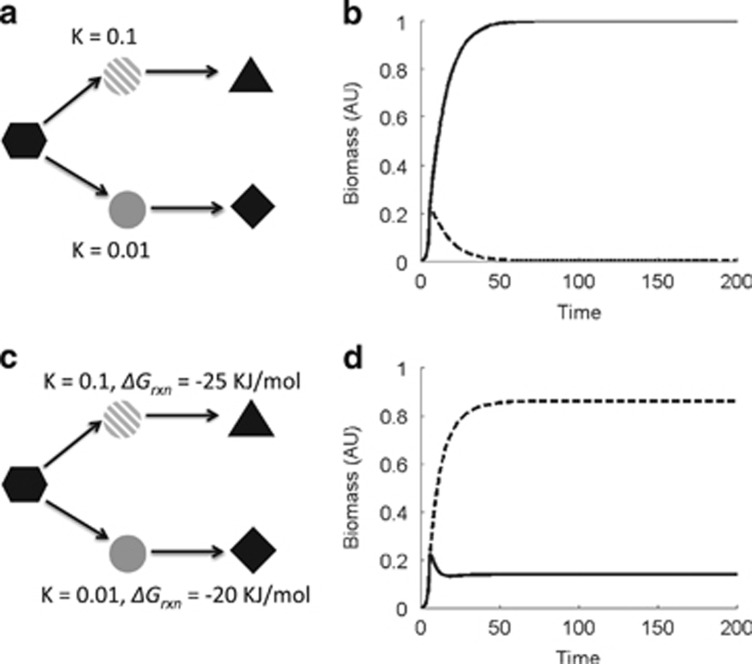
Population dynamics of two species living on a single substrate in a chemostat, modelled using an empirical, kinetic model (**a**, **b**) and a thermodynamic model (**c**, **d**) (see Materials and methods). Panels (**b** and **d**) show relative biomass of species *X*_1_ (dashed line), with *v*_max_=1, *K*=0.1 and 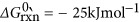 and species *X*_2_ (continuous line) with *v*_max_=1, *K*=0.01 and 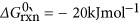. Total biomass is scaled to 1. Chemostat model parameters are *λ*=0.1 and *S*_0_=1000.

Using this thermodynamic growth model, we first consider the simplest and most idealised ecological case of two species living on a single substrate in a homogenous environment. This scenario can be realised in an ideal chemostat, where influx of substrate, dilution of metabolites and cells can be taken into account and modelled through appropriate ODEs (see Materials and methods). Chemostat models that consider microbial growth solely as a function of substrate uptake kinetics have been used to derive the exclusion principle; under the assumption of species having the same maximal growth rate and growth yield, there can only be one species existing in a chemostat with a single substrate and this species would be the one with the most favourable substrate uptake kinetics (Hsu *et al.*, 1977) (Figures 1a and b). When kinetic parameters of species are allowed to vary, there exists only a unique combination of maximal growth and kinetic uptake rates that would allow coexistence (Hsu *et al.*, 1977). When we model the scenario of two species with the same growth, uptake and yield parameters using the thermodynamic growth model, we find that coexistence of two species on a single substrate is a possible stable state, provided that these species produce different end products from the substrate (Figures 1c and d). This result can be understood simply by the fact that growth dynamics in the thermodynamic model is a function not only of yield and uptake parameters, but also of the free energy change available from the metabolic conversion. As free energy change of a specific reaction changes with the concentration of substrates and products (Equation 1), it dynamically affects the growth rate of each species according to the concentration of their metabolic products. In particular, the build-up of products reduces the free energy available for investing in growth rate. The result of this effect is similar to each species having a self-inhibition on their growth rate, which ultimately leads to the balancing of substrate consumption among different species producing different end products (Figure 1d). This results in a dynamical steady state, where the abundance of each species is governed by the free energy of the substrate–product pair (that is, catabolic metabolism) that they utilise. More specifically, we can show that for the simple case of two species living on a single substrate and assuming that all kinetic parameters governing substrate uptake, maximal growth rate and growth yield are equal, the ratio of species abundances at steady state is related to the 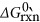 of their catabolic reactions (see Materials and methods and Equation 8). This result can be readily extended to multiple species coexisting on a single substrate within a chemostat, provided that they can utilise different and chemically feasible catabolic reactions.

### Thermodynamics-driven coexistence is most significant among conversions with low free energy change and such conversions are prevalent in nature

**Figure 2:**
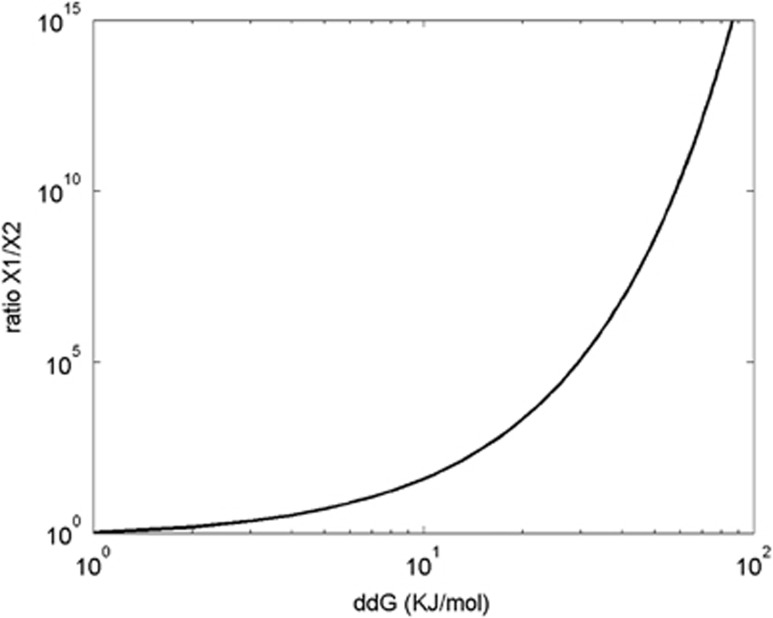
Double logarithmic plot showing the effect of thermodynamic energy difference (dΔG) on the steady-state biomass ratio of the two species *X*_1_ and *X*_2_ growing in a chemostat with shared substrate but differing waste products (according to Equation 8).

We explore what happens to species abundances as the difference in the 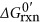 values between the two reactions that they utilise increases. As expected from Equation 8, this reveals that the ratio of the species abundances increases exponentially in favour of the species utilising the reaction with a higher change in free energy (more negative 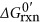) as the difference in 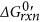 values increases (Figure 2). Thus, while coexistence due to thermodynamics is always possible theoretically, the abundance of species with metabolic reactions producing the smaller change in free energy becomes increasingly negligible as the differences in the free energy changes of the two reactions increase beyond ~50 kJ (mol Substrate)^−1^ (Figure 2). Here, it is important to note that the thermodynamic model we use ignores energy investments in anabolic reactions and maintenance and thus provides a conservative estimate of this energy range. Also, more complex biochemical reactions leading to the production of multiple products can increase the range of allowed coexistence (see Supplementary Text).

**Figure 3:**
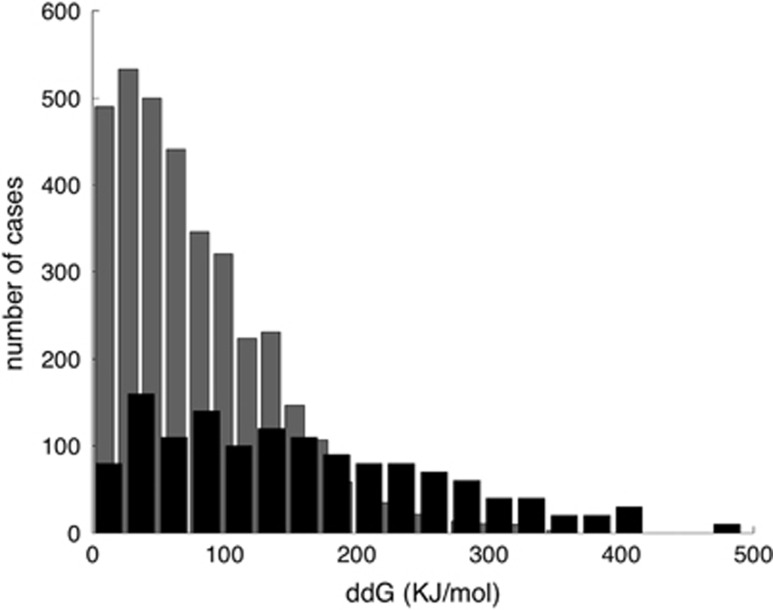
Histogram of the difference in thermodynamic energy available (dΔG°_Rxn_) between 85, algorithmically generated, stoichiometrically balanced anaerobic reactions starting from glucose (grey bars, see Materials and methods for details) and between all combinations of the reactions displayed in Table 1 (black bars, the case of glucose oxidation is excluded from these combinations and the number of the cases is scaled by 10 to fit the same graph as the data from algorithmic case).

Although Figure 2 provides a conservative estimate of the energy difference in catabolic reactions that would sustain coexistence of species at significant frequencies for the above reasons, it is still useful to consider the relevance of this energy regime in nature. In particular, are there chemically feasible catabolic reactions that are available for different species to utilise for the conversion of the same substrate, and that have 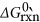 values within ~50 kJ (mol Substrate)^−1^ of each other? To answer this question, we collect the 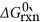 of known growth-supporting microbial catabolic reactions using tabulated free energy changes, with particular focus on microbial reactions occurring in anaerobic environments like sediments, animal guts and anaerobic digesters (Table 1) (Thauer *et al.*, 1977; Conrad *et al.*, 1986; Seitz *et al.*, 1988; Schink, 1997; Kleerebezem and Stams, 2000; Walker *et al.*, 2009; Dolfing, 2013; Worm *et al.*, 2014). As seen from Table 1, many of these reactions are within 20–100 kJ of each other (Figure 3). To further extend this analysis and account for any possible biases in the known metabolic conversions supporting microbial growth, we generated all chemically feasible reactions within a bounded search space. The aim was to get an ample set of reactions utilising glucose as a substrate in a fermentative manner (see Materials and methods). This exhaustive exploration of thermodynamically and stoichiometrically feasible metabolic conversions from glucose revealed that most of these reactions have 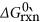 values within 50–100 kJ (mol Substrate)^−1^ of each other (Figure 3).

### An example of thermodynamics-driven coexistence among propionate degraders further illustrates product effects on coexistence

**Figure 4:**
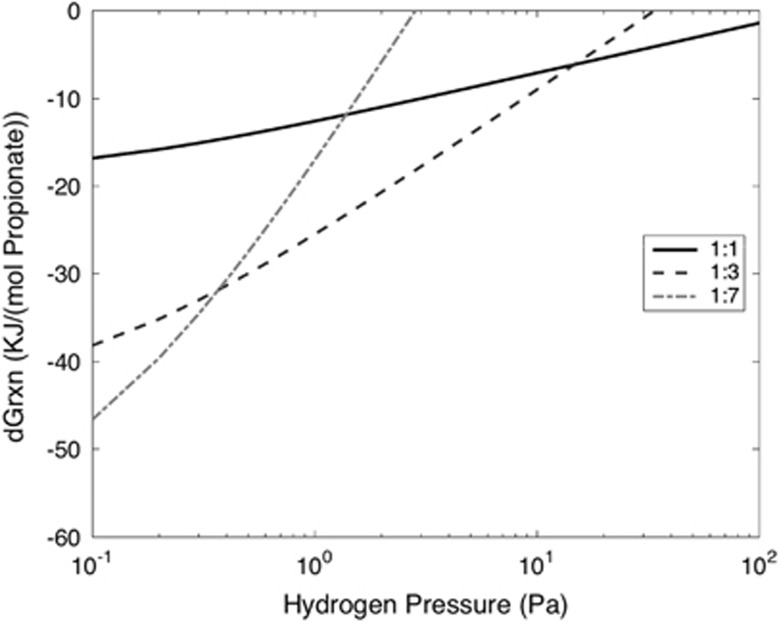
Thermodynamic energy of three propionate degrading reactions (from Table 1), normalised per propionate, at different ambient H_2_ pressures. Each reaction produces H_2_ as a byproduct, and the line type indicates the reaction stoichiometry between the propionate and hydrogen (as shown in the legend). The conditions used to calculate the reaction thermodynamics are pH=7, 1 mmol l^−1^ of ambient propionate, acetate and HCO_3_^−^ and varying ambient H_2_ pressures as shown on the x axis.

To further demonstrate the thermodynamic inhibition effect with an example, we consider three different anaerobic oxidation reactions for propionate (Table 1). Two of these reactions are identified to be taking place in anoxic paddy soils in a simultaneous manner (Gan *et al.*, 2012), indicating that the mediating microbes are coexisting in these environments. Although each reaction has a different standard thermodynamic energy change they also differ in the stoichiometry of their waste products, and in particular H_2_, produced. At standard conditions, that is, P(H_2_)=1 atm, none of the reactions is energy yielding. However, when applying more realistic conditions of milli molar concentrations of substrates and products, there is a H_2_ pressure at which any of the three reactions will be exothermic. Interesting here is that when we consider the full range of biologically feasible H_2_ pressures, we find that the reaction that can generate the highest amount of energy per propionate gets inhibited at a lower ambient H_2_ pressure than the other two reactions (Figure 4). This is underpinned by the stoichiometry of the reactions, where the reaction that can yield the highest energy at low H_2_ pressures has also the highest H_2_ yield (propionate:H_2_ stoichiometry=1:7). In other words, the thermodynamic advantage at low H_2_ pressures for the highest energy yielding reaction is removed by the rapid accumulation of H_2_ from its own reaction turnover. This example further highlights the fact that microbial growth will be dominated by the thermodynamics of the supporting reactions, which can then lead to new regimes of coexistence and diversity among competing reactions due to product accumulation.

### Thermodynamics effects dominate for low-energy metabolic conversions, but can be readily overridden by kinetic factors for high-energy metabolic conversions

**Figure 5:**
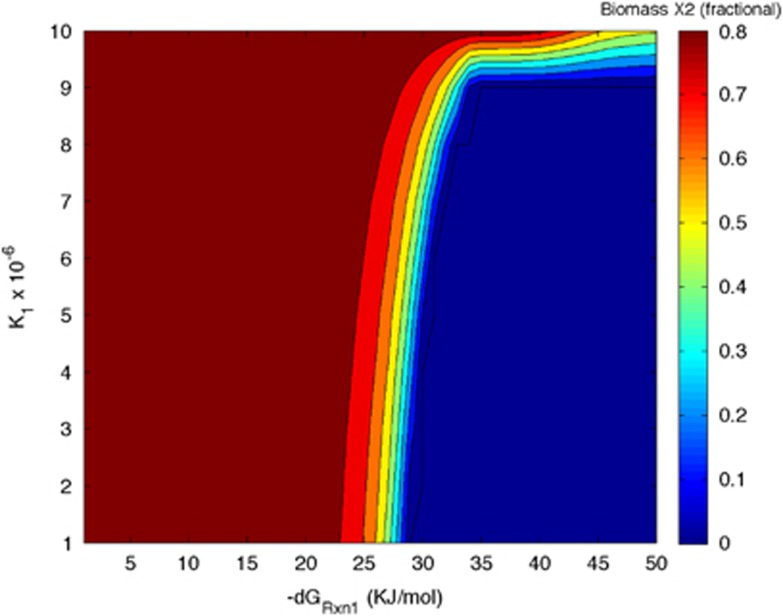
Numerical simulation showing the effect of substrate affinity (parameter *K* in Equation 2) and free energy values on the steady-state biomass composition. The colour mapping indicates biomass of species *X*_2_ as fraction of total biomass. The y axis shows the *K*_1_ of species *X*_1_, while *K*_2_ for species *X*_2_ is fixed at 10 × 10^−6^. The x axis shows the free energy of metabolic conversion reaction for species *X*_1_, while that of species *X*_2_ is always set to be 5 kJ (mol Substrate)^−1^ lower (i.e, thermodynamically more favourable) than that for species *X*_1_.

In the above analyses, we considered species coexistence solely due to differences in their metabolic conversions and resulting thermodynamic effects. In reality, species can diversify in many different traits governing their metabolism and growth. Among such traits, those relating to kinetic properties such as substrate uptake rates could be particularly relevant for the maintenance of species diversity as highlighted by the mathematical studies on the exclusion principle (Hsu *et al.*, 1977). To address this possibility, we re-consider the case of two species living on a single substrate by relaxing the assumption that these species have the same substrate uptake kinetics. Assuming a scenario where one species uses a slightly more energy-rich metabolic conversion, we consider alterations in the uptake kinetics of this species. In other words, we explore the possibility of kinetic effects counteracting the effects arising from thermodynamics in the context of species competition and coexistence. As expected, kinetic effects can counteract thermodynamic effects in a way such that a species utilising a more energy-rich metabolic conversion can become the rare species if it has weaker substrate uptake dynamics (Figure 5). We find that this counteracting effect of kinetics over thermodynamics becomes more dominant as the 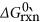 values of the metabolic conversions considered for the different species increases. This result can be understood directly from the thermodynamic growth model (Equation 2); as the 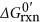 values increase, the thermodynamic effects arising from product accumulation become negligible and the growth dynamics are increasingly governed by substrate uptake kinetics (for further analyses, see Supplementary Text).

## Discussion

We have considered a thermodynamic model for microbial growth and analysed the ensuing population dynamics in the context of competition and coexistence. The model is derived from the fundamental principles of conservation of energy and thermodynamics in metabolic conversions fuelling microbial growth. The key result from this first-principles model is that utilisation of different metabolic conversions by different species can allow for their coexistence on a single substrate under a homogenous environment. We find that the abundance of different species under this circumstance is governed by the change in standard free energy of the reaction that they utilise, and that species utilising reactions that are within ~50 kJ (mol Substrate)^−1^ of the reaction with highest change in free energy would coexist at significant frequency. An analysis of known and chemically possible metabolic conversion reactions shows that this is a biologically relevant regime, where many biochemical reactions known to sustain microbial growth are found. This indicates that significant amounts of microbial diversity on a single substrate could have initially emerged from, or are being sustained by thermodynamic constraints.

As the change in standard free energy of the utilised metabolic conversion reactions increases, however, we find that kinetic effects such as differences in substrate uptake rates overcome the thermodynamics effects on microbial growth dynamics. As a result, it can be expected that any change in kinetic parameters (for example, by evolutionary change) would easily disrupt thermodynamic-driven diversity emerging under metabolic conversions with large change in free energy. The same is not true for metabolic conversions with small change in Gibbs free energy, where we find that species utilising metabolic pathways with 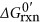 of −20 vs −25 kJ (mol Substrate)^−1^ can still coexist even with a 10-fold difference in their substrate half saturation constants (Figure 5). We emphasise that these predictions on the energy ranges leading to coexistence are conservative estimates, as the thermodynamic model used here considers that all of the free energy available from the metabolic conversion is invested into growth rate (Hoh and Cord-Ruwisch, 1996). In reality, some of this free energy would need to be invested in driving anabolic reactions and other cellular maintenance processes (Kleerebezem and Stams, 2000; Jin and Bethke, 2003; Rodríguez *et al.*, 2008). As a result, even metabolic conversions with higher free energy change could enter a regime of thermodynamic inhibition, and offer a window for the emergence of thermodynamics-driven diversity.

The presented model considers the thermodynamics of microbial growth, by considering a overall growth-supporting metabolic reaction (for example, glucose to acetate). In reality, cellular metabolism takes place over many reactions that finally reach a metabolic end product. Thus, the reaction Gibbs free energy change from the overall reaction is split among all the individual reactions and some of it needs to be invested to achieve an appropriate flux for these reactions (Flamholz *et al.*, 2013). The consequence of this is that not all of the Gibbs free energy change from the overall reaction can be invested in growth rate, as we assume here. Therefore, our estimates for the effects of thermodynamic inhibition could act in a larger parameter regime, that is, even for overall reactions with larger Gibbs free energies than studied here. In future work, it could be possible to consider different reaction pathways in different species to get a more accurate model of their thermodynamic growth dynamics. Indeed, studies in this direction are already being employed to compare different pathways (Flamholz *et al.*, 2013) and assess pathway feasibility under different conditions (González-Cabaleiro *et al.*, 2013; Cueto-Rojas *et al.*, 2015).

Although the thermodynamic constraints highlighted here act similar to inhibition driven by metabolic byproducts (De Freitas and Fredrickson, 1978; Lenski and Hattingh, 1986), it is important to note that the former does not inhibit microbial growth *per se*, but emerge from the drive towards chemical equilibrium in the given metabolic reaction sustaining growth. Therefore, each given species is specifically affected by the build-up of its own products. The inherent thermodynamic mechanism is thus to punish specifically the fastest growing organisms the earliest, and thereby favouring the coexistence of a high number of different metabolic conversions in the environment. The products of these diverse metabolic conversions can then be utilised by the same species or different ones as energy source, resulting in an example of ‘niche creation', and potentially leading to the emergence of further microbial diversity and interactions through adaptation. Indeed, anaerobic microbial communities are frequently characterized by abundance of such interlinked metabolic conversions (Schink, 1997, 2002).

The findings of this study suggest that microbial coexistence can readily arise under metabolic growth-supporting reactions with low free energy change. A direct experimental test for this proposition for this would be to grow two different species, or synthetically tagged variants of the same species, on a single substrate. The choice of the species and substrate should be such that each species can only utilise metabolic growth-supporting reactions with low free energy change. Such an experiment can be run under chemostat conditions, as well as under batch conditions that test mutual invasion from low frequency. Examples would include anaerobic growth on propionate or glycerol, where different metabolic pathways are known to exist. A broader suggestion from this study is that environments that mainly allow for growth-supporting metabolic reactions with low free energy change should harbour more metabolic diversity compared with environments that allow for metabolic reactions with high free energy change. This proposition could potentially be tested through the use of increasingly available metagenomics data from different environments. Examples for former type of environments would include anaerobic digesters, animal guts, wet soils and ocean sediments of highly productive regions. In these environments, high concentrations of substrates along with the lack of strong oxidising agents like oxygen or nitrate lead to accumulation of high concentrations of waste compounds. Thus, we expect microbial growth in these environments to be mainly limited by the lack of free energy available from the specific metabolic conversions a given species utilises. Microbes can overcome this limitation by evolving an ability to produce different waste products from the same substrate molecule. This way they can maintain growth by producing those products that are only present at very low concentrations in the environment, and overcoming any thermodynamic inhibition from accumulated products. This situation is analogous to a river flowing from the mountains to the sea, at its steeper parts the river is maintained in a single basin, while running over the flat surface close to the coast it starts to meander and form many little rivers in a delta.

## Figures and Tables

**Table 1:** Thermodynamic energy under standard conditions (ΔG°_Rxn_) for selected catabolic reactions

*Name*	*Reaction*	*dG° (KJ mol^−1^, pH 7)*	*Citation*
Glucose oxidation/Respiration	C_6_H_12_O_6_+6O_2_ −> 6HCO_3_^−^ +6H^+^	−2843.8	Thauer *et al.* (1977)^a^
Homoacetic fermentation of glucose	C_6_H_12_O_6_ −> 3C_2_H_3_O_2_^−^+3H^+^	−310.9	Thauer *et al.* (1977)^a^
Ethanol fermentation of glucose	C_6_H_12_O_6_+2H_2_O −> 2C_2_H_6_O+2HCO_3_^−^+2H^+^	−225.6	Thauer *et al.* (1977)^a^
Butyric acid fermentation of glucose	C_6_H_12_O_6_+2H_2_O −> 2HCO_3_^−^+2H^+^+2H_2_+C_4_H_7_O_2_^−^	−214.6	Thauer *et al.* (1977)^a^
Lactic acid fermentation of glucose	C_6_H_12_O_6_ −> 2 C_3_H_5_O_3_^−^+2H^+^	−198.2	Thauer *et al.* (1977)^a^
Pyruvate fermentation of glucose	C_6_H_12_O_6_ +2H_2_O −> C_3_H_3_O_3_^−^+C_2_H_3_O_2_^−^+HCO_3_^−^+3H^+^+3H_2_	−159	Thauer *et al.* (1977)^a^
Methanogenesis (hydrogenotrophic)	4H_2_+HCO_3_^−^+H^+^ −> CH_4_+3H_2_O	−135.6	Seitz *et al.* (1988)
Lactate oxidation	C_3_H_5_O_3_^−^+H_2_O −> C_2_H_3_O_2_^−^+2H_2_+CO_2_	−8.8	Walker *et al.* (2009)
Lactate oxidation with sulphate	C_3_H_5_O_3_^−^+H^+^+0.5SO_4_^2−^ −> 0.5H_2_S+H_2_O+CO_2_+C_2_H_3_O_2_^−^	−87.8	Thauer *et al.* (1977)^a^
Ethanol oxidation	C_2_H_6_O+H_2_O −> C_2_H_3_O_2_^−^+H_2_+H^+^	9.6	Thauer *et al.* (1977)^a^
Butyrate degradation	C_4_H_7_O_2_^−^+H_2_O −> 2C_2_H_3_O_2_^−^+H^+^+2H_2_	48.3	Rodríguez *et al.* (2008)
	C_4_H_7_O_2_^−^+2H_2_+H^+^ −> C_4_H_10_O+H_2_O	−56.4	Rodríguez *et al.* (2008)
Propionate degradation	2C_3_H_5_O_2_^−^+2H_2_O −> 3C_2_H_3_O_2_^−^+H^+^+2H_2_	48.4	Dolfing (2013)
	C_3_H_5_O_2_^−^+3H_2_O −> C_2_H_3_O_2_^−^+HCO_3_^−^+3H_2_+H^+^	76.5	Dolfing (2013)
	C_3_H_5_O_2_^−^+7H_2_O −> 3HCO_3_^−^+7H_2_+2H^+^	181.1	Thauer *et al.* (1977)
Glycolate degradation	C_2_H_3_O_3_^−^+H^+^+H_2_O −> 2CO_2_+3H_2_	19.3	Schink (1997)
Acetate degradation	C_2_H_3_O_2_^−^+H^+^+2H_2_O −> 2CO_2_+4H_2_	94.6	Schink (1997)
Methanogenesis (acetoclastic)	C_2_H_3_O_2_^−^+H_2_O −> CH_4_+HCO_3_^−^	−31.1	Schink (1997)

a Computed according to listed formation energies.
