# Supplementary material for: Microbial diversity arising from thermodynamic constraints
Source: ISME J. 2016 Apr 1;10(11):2725–33. doi: 10.1038/ismej.2016.49 (PMC5042319; doi:10.1038/ismej.2016.49)
Supplement: Supplementary Information [file ismej201649x1.pdf]

Microbial diversity arising from thermodynamic constraints

Tobias Großkopf & Orkun S. Soyer

Supplementary Text

<u>Contents:</u>

1. Model of a two-species, single substrate chemostat. 2

1.1. Case without thermodynamic inhibition…………………………… 2

1.2. Case with thermodynamic inhibition……………………………….. 3

2. The thermodynamic case with a generalized growth reaction that has many end-products. 7

3. The thermodynamic case with alternative thermodynamic growth formulations. 10

4. The effects of model parameters on species co-existence 12

5. Supplementary Figures 13

1. Model of a two-species, single substrate chemostat.

We consider two species X_1 and X_2 in a chemostat, where they are consuming the same substrate, S. Species X_1 and X_2 are assumed to process the substrate through different metabolic pathways to produce different products, P_1 and P_2 respectively. The feed-in rates of the species to the chemostat is considered to be zero, while the feed-in rate of the substrate is given by S_0. The dilution rate (per hour) of the chemostat is considered to be λ.

Using these notations, we can construct ordinary differential equations (ODEs) to model the dynamics in a chemostat as shown below. In doing so, we consider two different cases.

1.1. Case without thermodynamic inhibition. Under this case we assume that thermodynamics is not relevant for describing the growth dynamics of each species. This is the generally considered case and the equations we describe below are used in numerous studies modeling both chemostat and batch systems and microbial dynamics in these. We describe microbial growth by the Monod term (Monod, 1949);

$$v = \frac{v_{\max} \cdot [S]}{K + [S]} \qquad \textit{Eq. S1}$$

where K is the so-called half-saturation constant and v_{max} is the maximum substrate uptake rate possible. With the growth rate of each species given by *Eq. S1*, we write the ODEs describing species and metabolite dynamics in the two species – one substrate chemostat as follows;

$$\begin{aligned}
\frac{d[S]}{dt} &= \left([S_0]-[S]\right)\cdot \lambda - [X_1]\cdot v_1 - [X_2]\cdot v_2 \\
\frac{d[X_1]}{dt} &= Y_1 \cdot [X_1]\cdot v_1 - \lambda \cdot [X_1] \\
\frac{d[X_2]}{dt} &= Y_2 \cdot [X_2]\cdot v_2 - \lambda \cdot [X_2] \\
\frac{d[P_1]}{dt} &= [X_1]\cdot v_1 - \lambda \cdot [P_1] \\
\frac{d[P_2]}{dt} &= [X_2]\cdot v_2 - \lambda \cdot [P_2]
\end{aligned} \qquad \textit{Eq. S2}$$

, where v_i represents the growth rate of species i and is given by the generic form shown in *Eq. S1*. These ODEs describe the temporal dynamics of the species and metabolites in the chemostat. Of particular interest is the steady state, where each of the differential equations is equal zero, i.e. none of the species and metabolite concentrations change. To understand the species concentrations at steady state, we can solve *Eq. S2*. by setting the left side of each of the ODEs equal to zero. In particular, we can use the steady state condition for the second and third ODEs to derive;

$$\lambda \cdot \left[\overline{X_1}\right] = Y_1 \cdot \left[\overline{X_1}\right] \cdot v_1$$
$$\lambda \cdot \left[\overline{X_2}\right] = Y_2 \cdot \left[\overline{X_2}\right] \cdot v_2 \qquad \textit{Eq. S3}$$

where the bar notation indicates steady state values. We notice that *Eq. S3* can only be satisfied if the species concentrations at steady state are zero (i.e. the species is not maintained in the chemostat), or when their steady state growth rates times their yield factor are equal to the dilution rate, i.e.;

$$\lambda = Y_1 \cdot v_1$$
$$\lambda = Y_2 \cdot v_2 \qquad \textit{Eq. S4}$$

Fulfillment of this condition is only possible for a specific combination of parameter values. Outside of these parameters the above treatment leads to the formulation of the exclusion principle on a single substrate, whereby only one species dominates in the chemostat and the other one is washed out (Hsu *et al.*, 1977) (see also Figure S3).

1.2. Case with thermodynamic inhibition. Under this case we consider the thermodynamics of metabolic conversion and utilize a previously described thermodynamic model for microbial growth that considers a reversible enzymatic reaction as a proxy for the microbial-growth supporting metabolism (Hoh & Cord-Ruwisch, 1996). The growth rate is given by;

$$v = \frac{v_{\max} \cdot [S] \cdot \left(1 - \exp\left(\Delta G_r\right)\right)}{K + [S] \cdot (1 + k_r \cdot \exp(\Delta G_r))} \qquad \textit{Eq. S5}$$

where the constants k_r and K are composite parameters representing the ratio of the forward and backward reaction rates and substrate turnover rate respectively, and ΔG_{rxn} is the thermodynamic energy available in the reversible reaction for a given set of substrate ([S]) and product ([P]) concentrations. This energy can be calculated from the standard reaction free energy, using;

$$\Delta G_{rxn} = \Delta G_{rxn}^0 + R \cdot T \cdot \ln\left(\frac{\prod_i [P]_i^{n_i}}{\prod_j [S]_j^{m_j}} \right) \qquad \textit{Eq. S6}$$

where the multiplications indexed by i and j are over the products and substrates participating in the reaction respectively, n_i and m_j are the chemical stoichiometric coefficients of products and substrates respectively, R and T are the gas constant and the absolute temperature in Kelvin respectively, and ΔG_{rxn}^0 is given by the difference in the free energies of formation of products and substrates.

For the simple case of two species utilising the same substrate S in a chemostat and producing a different waste product, the ODEs describing species and metabolite dynamics is as before, given in *Eq. S2*, and *Eq. S4* still holds. Substituting the new growth rate into this equation, we get;

$$\begin{aligned} \lambda \cdot \left[\overline{X_1}\right] &= Y_1 \cdot \left[\overline{X_1}\right] \cdot \left(\frac{v_{\max,1} \cdot [\overline{S}] \cdot \left(1 - \exp\left(\Delta G_{rxn,1}\right)\right)}{K_1 + [\overline{S}] \cdot (1 + k_{r,1} \cdot \exp(\Delta G_{rxn,1}))} \right) \\ \lambda \cdot \left[\overline{X_2}\right] &= Y_2 \cdot \left[\overline{X_2}\right] \cdot \left(\frac{v_{\max,2} \cdot [\overline{S}] \cdot \left(1 - \exp\left(\Delta G_{rxn,2}\right)\right)}{K_2 + [\overline{S}] \cdot (1 + k_{r,2} \cdot \exp(\Delta G_{rxn,2}))} \right) \end{aligned} \qquad \textit{Eq. S7}$$

To simplify equation *S7*, we can assume that parameters K_1 and K_2 are much larger compared to the second term in the denominator and thus, this term can be ignored. This assumption corresponds to either or both the steady state concentration of the substrate and the term $(1 + k_r \cdot \exp(\Delta G))$ being very small compared to the substrate turnover rate. A small value of steady state concentration of the substrate would be inline with the high turnover rate, while a small value of k_r would mean that the

backward rate is large compared to forward rate in the assumed reversible metabolic reaction, i.e. we have considerably backward flux. Under this assumption of high substrate turn-over rate, we can re-arrange equation *S7* to derive a condition for the steady state as;

$$
\begin{aligned}
&Y_2 \cdot \left(\frac{v_{\max,2} \cdot \overline{[S]} \cdot \left(1 - \exp\left(\Delta G_{rxn,2}\right)\right)}{K_2} \right) = Y_1 \cdot \left(\frac{v_{\max,1} \cdot \overline{[S]} \cdot \left(1 - \exp\left(\Delta G_{rxn,1}\right)\right)}{K_1} \right) \\
&\frac{Y_2 \cdot K_1 \cdot v_{\max,2}}{Y_1 \cdot K_2 \cdot v_{\max,1}} = \frac{1 - \exp\left(\Delta G_{rxn,1}\right)}{1 - \exp\left(\Delta G_{rxn,2}\right)} \\
&A \cdot \left(1 - \exp\left(\Delta G_{rxn,2}\right)\right) = 1 - \exp\left(\Delta G_{rxn,1}\right) \qquad \textit{Eq. S8} \\
&A - 1 = A \cdot \exp\left(\Delta G_{rxn,2}\right) - \exp\left(\Delta G_{rxn,1}\right)
\end{aligned}
$$

where A is a composite parameter given by $Y_2 \cdot v_{\max.2} \cdot K_1 / Y_1 \cdot v_{\max.1} \cdot K_2$. Thus, steady state condition can be satisfied with the two species coexisting, for the correct combination of their kinetic parameters and metabolic free energies. Since metabolic free energies are a function of substrate and product concentrations, coexistence is possible in a larger dynamical regime compared to the kinetics-only model (see also Figure S3). To get a sense on how species frequencies at steady state depend on the concentrations of their metabolic end-products, we can make the simplifying assumption that yield, maximal growth, and uptake parameters of the two species are the same, i.e. A=1; leading to $\exp(\Delta G_{rxn,1}) = \exp(\Delta G_{rxn,2})$. Note that from an evolutionary perspective, this assumption would be likely to hold right after a speciation event. In the case of A=1, and assuming each species only produces one product, we can substitute *Eq. S6* into the simplified *Eq. S8* to derive a relation between the end product concentrations at steady state as a function of the standard free energy of the metabolic conversions;

$$\exp\left(\Delta G_{rxn,1}\right) = \exp\left(\Delta G_{rxn,2}\right)$$

$$\Delta G^0_{rxn,1} + R\cdot T\cdot \ln\left(\frac{\left[\overline{P_1}\right]}{\left[\overline{S}\right]}\right) = \Delta G^0_{rxn,2} + R\cdot T\cdot \ln\left(\frac{\left[\overline{P_2}\right]}{\left[\overline{S}\right]}\right)$$

$$\Delta G^0_{rxn,2} - \Delta G^0_{rxn,1} = R\cdot T\cdot \left(\ln\left(\frac{\left[\overline{P_1}\right]}{\left[\overline{S}\right]}\right) - \ln\left(\frac{\left[\overline{P_2}\right]}{\left[\overline{S}\right]}\right)\right) \qquad \textit{Eq. S9}$$

$$\frac{\Delta G^0_{rxn2} - \Delta G^0_{rxn,1}}{R\cdot T} = \ln\left(\frac{\left[\overline{P_1}\right]}{\left[\overline{P_2}\right]}\right)$$

$$\exp\left(\frac{\Delta G^0_{rxn,2} - \Delta G^0_{rxn,1}}{R\cdot T}\right) = \frac{\left[\overline{P_1}\right]}{\left[\overline{P_2}\right]}$$

Thus, when the two species in the chemostat co-exist at steady state, their metabolic byproduct will do so as well. Further, the steady state concentrations of these products will show a specific ratio that reflects the difference in the Gibbs free energy of formation of the corresponding metabolic pathways leading from the substrate to these products.

Using the second and fourth (or third and fifth) ODEs shown in *Eq. S2*, and utilizing the fact that at steady state, growth rate of each species needs to be equal to the other one and the dilution rate, we can show that the concentration of the species would relate to the concentrations of the metabolic end-products;

$$\lambda\cdot\left[\overline{X_1}\right] = Y_1\cdot\left[\overline{X_1}\right]\cdot v_1$$

$$Y_1 = \lambda/v_1$$

$$\lambda\cdot\left[\overline{P_1}\right] = \left[\overline{X_1}\right]\cdot v_1 \qquad \textit{Eq. S10}$$

$$\overline{[P_1]} = \left[\overline{X_1}\right]/Y_1$$

Thus, the ratio of the species concentrations will also obey the relation given in *Eq. S9,* adjusted by the yield constants for the two species.

The result of this mathematical treatise is that under the consideration of thermodynamics, two species consuming the same substrate can co-exist in a

chemostat as long as they utilize two different metabolic pathways leading to two different metabolic byproducts and thus giving rise to different Gibbs free energy of reaction. The relative ratio of the concentrations of the species in the chemostat will reflect the difference between these free energies, with the species utilizing the reaction with the largest negative value of ΔG_{rxn} displaying the highest concentration.

2. The thermodynamic case with a generalized growth reaction that has many end-products.

We can extend the above treatise readily to a general case of just two species, but utilizing a generalized overall growth reaction that results in many different end-products. This would correspond to a case, where a species utilizes mixed fermentation as seen for example in *Lactobacillus* species (or many other fermenting bacteria). For such multi-product conversions, the above treatise would remain unchanged up to, and including *Eq. S8*. Making the same assumption of *A*=1, as before, the further simplification of this equation would still lead to $\exp\left(\Delta G_{rxn,1}\right)=\exp\left(\Delta G_{rxn,2}\right)$, but now these thermodynamic terms would be defined in a general form, with many end-products. Thus, *Eq. S9* would become;

$$\exp\left(\Delta G_{rxn,1}\right)=\exp\left(\Delta G_{rxn,2}\right)$$

$$\Delta G^0_{rxn,1}+R\cdot T\cdot\ln\left(\frac{\prod_i\left(\overline{[P_i]}\right)^{\alpha_i}}{\left[\bar{S}\right]}\right)=\Delta G^0_{rxn,2}+R\cdot T\cdot\ln\left(\frac{\prod_j\left(\overline{[P_j]}\right)^{\beta_j}}{\left[\bar{S}\right]}\right) \qquad \textit{Eq. S11}$$

where the indices *i* and *j* are over the end-products and the exponents α_i and β_j are the stoichiometric factors for these products, respectively for species 1 and 2. We can solve this equation to get a similar result as before;

$$\Delta G^0_{rxn,1} + R\cdot T\cdot \ln\left(\frac{\prod_i \left(\overline{[P_i]}\right)^{\alpha_i}}{\left[\bar{S}\right]}\right) = \Delta G^0_{rxn,2} + R\cdot T\cdot \ln\left(\frac{\prod_j \left(\overline{[P_j]}\right)^{\beta_j}}{\left[\bar{S}\right]}\right)$$

$$\Delta G^0_{rxn,2} - \Delta G^0_{rxn,1} = R\cdot T\cdot \ln\left(\frac{\prod_i \left(\overline{[P_i]}\right)^{\alpha_i}}{\left[\bar{S}\right]}\right) - R\cdot T\cdot \ln\left(\frac{\prod_j \left(\overline{[P_j]}\right)^{\beta_j}}{\left[\bar{S}\right]}\right) \qquad \textit{Eq. S12}$$

$$\frac{\Delta G^0_{rxn,2} - \Delta G^0_{rxn,1}}{R\cdot T} = \ln\left(\frac{\prod_i \left(\overline{[P_i]}\right)^{\alpha_i}}{\prod_j \left(\overline{[P_j]}\right)^{\beta_j}}\right)$$

$$\exp\left(\frac{\Delta G^0_{rxn,2} - \Delta G^0_{rxn,1}}{R\cdot T}\right) = \frac{\prod_i \left(\overline{[P_i]}\right)^{\alpha_i}}{\prod_j \left(\overline{[P_j]}\right)^{\beta_j}}$$

This equation relating reaction Gibbs free energies to steady state end-product concentrations can again be related to species concentrations. In this generalized case, we have similar ODEs as shown in *Eq. S2*, but there would be a larger number of equations describing the rate of change for each of the end-products;

$$\frac{d[P_{1,1}]}{dt} = \alpha_1\cdot\left[X_1\right]\cdot v_1 - \lambda\cdot\left[P_{1,1}\right], \ldots\ldots, \frac{d[P_{1,i}]}{dt} = \alpha_i\cdot\left[X_1\right]\cdot v_1 - \lambda\cdot\left[P_{1,i}\right]$$

$$\frac{d[P_{2,1}]}{dt} = \beta_1\cdot\left[X_2\right]\cdot v_2 - \lambda\cdot\left[P_{2,1}\right], \ldots\ldots, \frac{d[P_{2,j}]}{dt} = \beta_j\cdot\left[X_2\right]\cdot v_2 - \lambda\cdot\left[P_{2,j}\right] \qquad \textit{Eq. S13}$$

Thus, the relation previously given in *Eq. S10* would become;

$$\overline{[P_{1,1}]} = \alpha_1\cdot\overline{\left[X_1\right]}/Y_1, \ldots\ldots \overline{[P_{1,i}]} = \alpha_i\cdot\overline{\left[X_1\right]}/Y_1$$

$$\overline{[P_{2,1}]} = \beta_1\cdot\overline{\left[X_2\right]}/Y_2, \ldots\ldots \overline{[P_{2,j}]} = \beta_j\cdot\overline{\left[X_2\right]}/Y_2 \qquad \textit{Eq. S14}$$

Putting this relation back into *Eq. S12*, we get;

$$\exp\left(\frac{\Delta G^0_{rxn,2} - \Delta G^0_{rxn,1}}{R \cdot T}\right) = \frac{\prod_i \left(\overline{[P_i]}\right)^{\alpha_i}}{\prod_j \left(\overline{[P_j]}\right)^{\beta_j}}$$

$$\exp\left(\frac{\Delta G^0_{rxn,2} - \Delta G^0_{rxn,1}}{R \cdot T}\right) = \frac{\prod_i \left(\alpha_i \cdot \left[\overline{X_1}\right] / Y_1\right)^{\alpha_i}}{\prod_j \left(\beta_j \cdot \left[\overline{X_2}\right] / Y_2\right)^{\beta_j}} \qquad \textit{Eq. S15}$$

In the simplest case, when $Y_1 = Y_2$ and $\alpha_i = \beta_j = 1$ (i.e. only one moles of each different product produced per one mol of substrate), and the only difference is the number of different products that are produced by species X_1 and X_2, *Eq. S15* simplifies to:

$$\exp\left(\frac{\Delta G^0_{rxn,2} - \Delta G^0_{rxn,1}}{R \cdot T}\right) = \frac{\left(\left[\overline{X_1}\right]\right)^i}{\left(\left[\overline{X_2}\right]\right)^j} \qquad \textit{Eq. S16}$$

This shows that the relation between steady state concentrations of the two species still relates to the difference in their reaction Gibbs free energies, but also to the number of their end products. Using $i = 1$ and $j = 2$ as an illustrative case, we can see that the relation between the steady state species concentrations turns into a power function. This means that only if the steady state concentration of the second species is one, there is no effect of the increased number of metabolic end-products it forms. If the steady state concentration of this species is above this value, having a higher number of end-products would be detrimental to its steady state frequency. Below that concentration an increased number of end-products would be beneficial. This makes intuitive sense, since the concentration of species is linked here to the concentrations of the end-products it produces. Figure S1 illustrates this relation further, where we use *Eq. S16* to plot the steady state concentration of one species, given the other one.

To conclude, the generalized case of multiple metabolic end-products still results in the steady state ratio of the species concentrations relating to the difference in the Gibbs free energy of their overall growth reactions. However, in this case the steady state frequencies of the species becomes a complex function of the number of their metabolic end-products. For example, it is possible for an energetically disfavored species to attain a higher steady state frequency, if the energetically favored species utilized multiple pathways leading to multiple end-products (e.g. assuming Y_1=Y_2, and

setting the free Gibbs energies so that reaction 1 is more energetically favorable, the same energy difference would lead to a lower $[X_1]/[X_2]$ as the i / j is increased). Note that this result is based on the assumption that by increasing its number of metabolic end-products, a species does not improve its overall reaction energetics. Here is an example: Assuming that the left side of *Eq. S14* was 100, we would get $[X_1] = 100$ and $[X_2] = 1$ with $i = j = 1$, but $[X_1] = 10$ and $[X_2] = 1$ with $i = 2$, and $j = 1$. For the latter case to maintain the original species concentrations of $[X_1] = 100$ and $[X_2] = 1$, the left side of the equation would have to increase from 100 to 10000, i.e. the energy difference would have to increase by approximately 2-fold.

3. The thermodynamic case with alternative thermodynamic growth formulations.

The efforts to provide a microbial growth model based on thermodynamic considerations can be grouped into distinct general categories. Particularly, these models are either based on a phenomological model of microbial metabolism, that considers connection between the energetics of catabolic and anabolic metabolism (Kleerebezem & Stams, 2000; Rodríguez *et al.*, 2008), or are derived from considering microbial growth as a single or series of reversible reactions (Hoh & Cord-Ruwisch, 1996; Jin & Bethke, 2003; Curtis, 2003; Liu *et al.*, 2001) and utilizing results from equilibrium dynamics of such reactions (Boudart, 1976). From a mathematical perspective, these two classes of models have a highly similar structure and result in the adjustment of the kinetic rate (based on substrate uptake) with a thermodynamic factor (Jin & Bethke, 2007). The differences in the models arise in the calculation of the free energy associated with this thermodynamic factor, with some models considering the full extent of the free energy available from the catabolic reaction to be invested in driving the growth rate (Curtis, 2003; Liu *et al.*, 2001; Hoh & Cord-Ruwisch, 1996), and others considering fractions of it invested in ATP production (i.e. anaobolic metabolism) and cell maintenance (Jin & Bethke, 2003; Kleerebezem & Stams, 2000; Rodríguez *et al.*, 2008).

The analysis in sections 1 and 2 considered a model from the former group of models, considering full energetic investment from the growth-supporting reaction into growth rate. From the mathematical treatise given above it is clear that our general conclusions with regards to co-existence depending on reaction Gibbs free energies

will not change with the alternative growth models that consider some of these energies invested in anabolic reactions. To make this point more tractable, we consider here the thermodynamic growth model developed by Kleerebezem and colleagues (Kleerebezem & Stams, 2000; Rodríguez *et al.*, 2008). The few other models in this category, in particular the one by Qusheng and Bethke (Jin & Bethke, 2003, 2007) would give similar results as those derived below.

The particular thermodynamic growth model considered by Kleerebezem and colleagues (Rodríguez *et al.*, 2008) gives the growth rate as a function of anabolic and catabolic reaction energetics;

$$v = \frac{1}{-\Delta G_{anab}} \cdot \left[q_s \cdot (1 - f_{dis}) \cdot \Delta G_{catab} - m_G \right] \qquad \textit{Eq. S17}$$

where ΔG_{anab} and ΔG_{catab} express the Gibbs free energies assumed to be invested in anabolic and catabolic metabolism respectively, q_s is the substrate uptake rate, f_{dis} is the fraction of the catabolic Gibbs energy that is assumed to be dissipated to establish a flux through the catabolic enzyme system, and m_G is the Gibbs energy dissipation rate for growth-independent maintenance purposes (Rodríguez *et al.*, 2008). In the application of this growth model, ΔG_{anab}, f_{dis}, and m_G are assumed to be fixed (determined empirically for a given species), ΔG_{catab} is calculated as described in *Eq. S6*, and q_s is calculated using;

$$q_s = q_s^{\max} \cdot \left[1 - \exp\left(f_{dis} \cdot \Delta G_{catab} \right) \right] \qquad \textit{Eq. S18}$$

where $q_s^{\max}$ is the maximal substrate uptake rate, analogous to v_{max} from section 1. Putting this term into *Eq. S17* and re-arranging, we can write the overall thermodynamic growth rate model as;

$$v = q_s^{\max} \cdot \frac{\Delta G_{catab}}{-\Delta G_{anab}} \cdot \left[1 - (1 - f_{dis}) \cdot \exp\left(f_{dis} \cdot \Delta G_{catab} \right) - f_{dis} \right] + \frac{m_G}{\Delta G_{anab}} \qquad \textit{Eq. S19}$$

We can analyse the original chemostat model presented in section 1 using this alternative model. We note that the analysis of steady state condition up to the derivation of *Eq. S4* would remain unaltered. The resulting condition given in *Eq. S7*

and *Eq. S8* would need to be re-written using the growth rate given in *Eq. S19*, resulting in;

$$Y_1 \cdot \left[q_{s,1}^{\max} \cdot \frac{\Delta G_{catab,1}}{-\Delta G_{anab,1}} \cdot \left[1-(1-f_{dis,1})\cdot \exp\left(f_{dis,1}\cdot \Delta G_{catab,1}\right) - f_{dis,1}\right] + \frac{m_{G,1}}{\Delta G_{anab,1}} \right] = Y_2 \cdot \left[q_{s,2}^{\max} \cdot \frac{\Delta G_{catab,2}}{-\Delta G_{anab,2}} \cdot \left[1-(1-f_{dis,2})\cdot \exp\left(f_{dis,2}\cdot \Delta G_{catab,2}\right) - f_{dis,2}\right] + \frac{m_{G,2}}{\Delta G_{anab,2}} \right] \quad \textit{Eq. S20}$$

This equality can be simplified by assuming all empirically determined parameters for the two species to be the same (i.e. $q_{s,1}^{\max} = q_{s,2}^{\max}$, $Y_1 = Y_2$, $m_{G,1} = m_{G,2}$, $\Delta G_{anab,1} = \Delta G_{anab,2}$, and $f_{dis,1} = f_{dis,2} = f_{dis}$). In this case, we have;

$$\Delta G_{catab,1} \cdot \left[1-(1-f_{dis})\cdot \exp\left(f_{dis}\cdot \Delta G_{catab,1}\right) - f_{dis}\right] = \Delta G_{catab,2} \cdot \left[1-(1-f_{dis})\cdot \exp\left(f_{dis}\cdot \Delta G_{catab,2}\right) - f_{dis}\right] \quad \textit{Eq. 21}$$

which further simplifies to;

$$\frac{\Delta G_{catab,1}}{\Delta G_{catab,2}} = \frac{(1-\exp\left(f_{dis}\cdot \Delta G_{catab,2}\right))}{(1-\exp\left(f_{dis}\cdot \Delta G_{catab,1}\right))} \quad \textit{Eq. 22}$$

While this equation is not readily solvable to obtain a functional form relating the Gibbs free energies associated with the catabolic reactions of each species to the steady state metabolic end-product and species concentrations, we show through numerical simulation that it results in the same relation as given by *Eq. S9* and *Eq. S10* and shown in Figure 2 (see Figure S2). We find that the relation deviates from that given by *Eq. S9* and *Eq. S10*, when the fraction of the catabolic Gibbs energy that each species invest into metabolic flux (i.e. $f_{dis,1}$ and $f_{dis,2}$) are different from each other. We conclude that the main effect on co-existence by considering this alternative model arises from the consideration of energy investment in anabolic reactions.

4. The effects of model parameters on species co-existence

Here, we first demonstrate with numerical simulations, how inclusion of thermodynamics leads to co-existence under a larger parameter regime. Particularly,

we consider the range of co-existence against the key chemostat parameters – the dilution and substrate feeding rates - and under the "kinetic-only" and thermodynamic models. This analysis shows that co-existence is possible in the kinetic-only model only for a specific combination of kinetic parameters (as shown previously (Hsu *et al.*, 1977)), resulting in a single line of co-existence in this plot (Figure S3C and S3D). We find that this line expands to a large region under the thermodynamic model (Figure S3A and S3B), as expected from the presented mathematical results.

Next, we analyse how the co-existence range in the thermodynamic model is affected by the different parameters of the model. To this end, we vary all parameters of the thermodynamic model against each other, to assess which of the parameters have the strongest effect on the co-existence (Figure S4). While most parameter combinations still lead to co-existence, we find that the reaction Gibbs free energy change has the most significant effect on co-existence.

5. Supplementary Figures

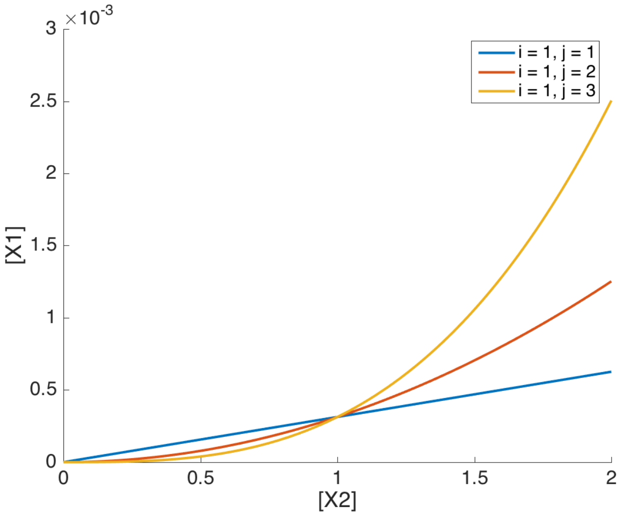

Figure S1. Effect of multiple metabolic end-products on species coexistence. The steady state concentration of one species was calculated given the other one and using *Eq. S16.* The other parameters used are; $dG°_{rxn1}$ = -100, $dG°_{rxn2}$ = -80 and the parameters i and j as shown on the graph legend.

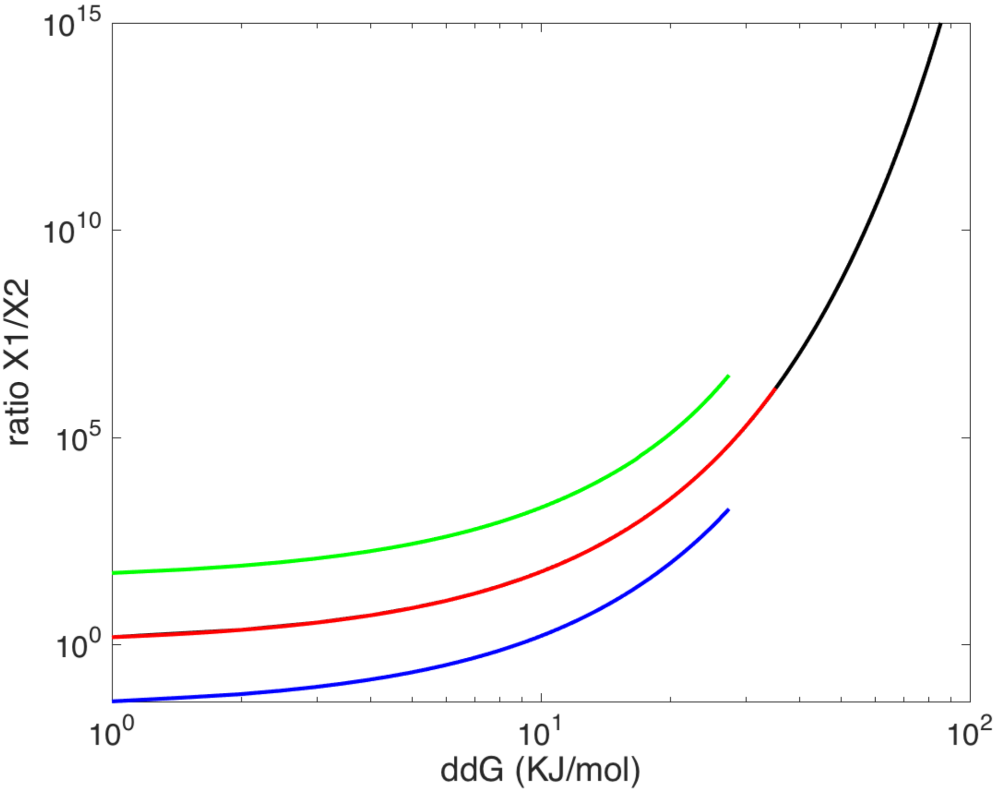

Figure S2. Effect of increasing difference in Gibbs free energy change on species frequencies, using the alternative thermodynamic model given in *Eq. S19*. The y-axis shows the steady state concentration ratio between the two species, while the x-axis shows the energy difference between their reaction Gibbs free energy change (*ddG*). The model was simulated considering $dG°_{rxn1} = dG°_{rxn2} + ddG$, and using the parameters; $dG°_{rxn2}$ = -25, $1/\Delta G_{anab}$ = 0.033 (based on (Heijnen & Van Dijken, 1992), m_G = 0.08 (based on (Rodríguez *et al.*, 2008)), q_{max} = 20e-3, and K = 1e-6 for substrate uptake function (given by a classical saturation function; q_{max}*[S]/(K+[S])). The dilution and substrate feed parameters for the chemostat were set to d = 0.01 and S_0 = 0.25. The curves shown in red, green, and blue result from simulations using $f_{dis,1} = f_{dis,2}$ = 0.35, $f_{dis,1}$ = 0.35, $f_{dis,2}$ = 0.5, and $f_{dis,1}$ = 0.5, $f_{dis,2}$ = 0.35, respectively. The black line shows the results from *Eq. S9* as shown in Figure 2. The green, red, and blue curves are given only up to a certain value of *ddG* due to instabilities in the numerical simulations (i.e. numerical integration failing to reach a stable steady state).

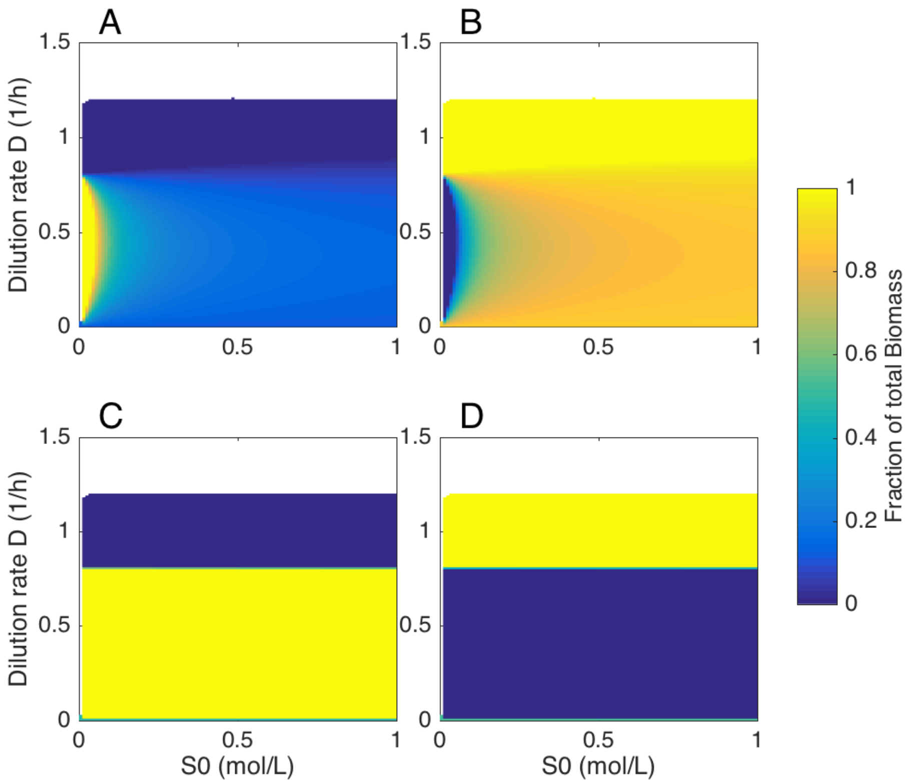

Figure S3. The fraction of steady state species concentrations in the chemostat (as shown in color legend) for different combinations of dilution rate (D, called λ in the equations) and feed substrate concentration (S_0). Panels A and C show the fractional biomass of species X_1, while panels B and D show the concentration of species X_2. The other model parameters used are: $V_{max,1}$ = 1, K_1 = 1e-4, dG_1 = -20, $V_{max,2}$ = 1.2, K_2 = 2e-4, dG_2 = -25, Y_1 = Y_2 = 1. Note the co-existence is possible in a wide for the thermodynamic model (A, B), whereas it is possible only at a specific dilution rate for the classical model (C,D) and under the parameter condition

$$K_1 \Big/ \left(Y_1 \cdot \frac{V_{max1}}{\lambda} - 1 \right) = K_2 \Big/ \left(Y_2 \cdot \frac{V_{max2}}{\lambda} - 1 \right)$$

(Hsu *et al.*, 1977).

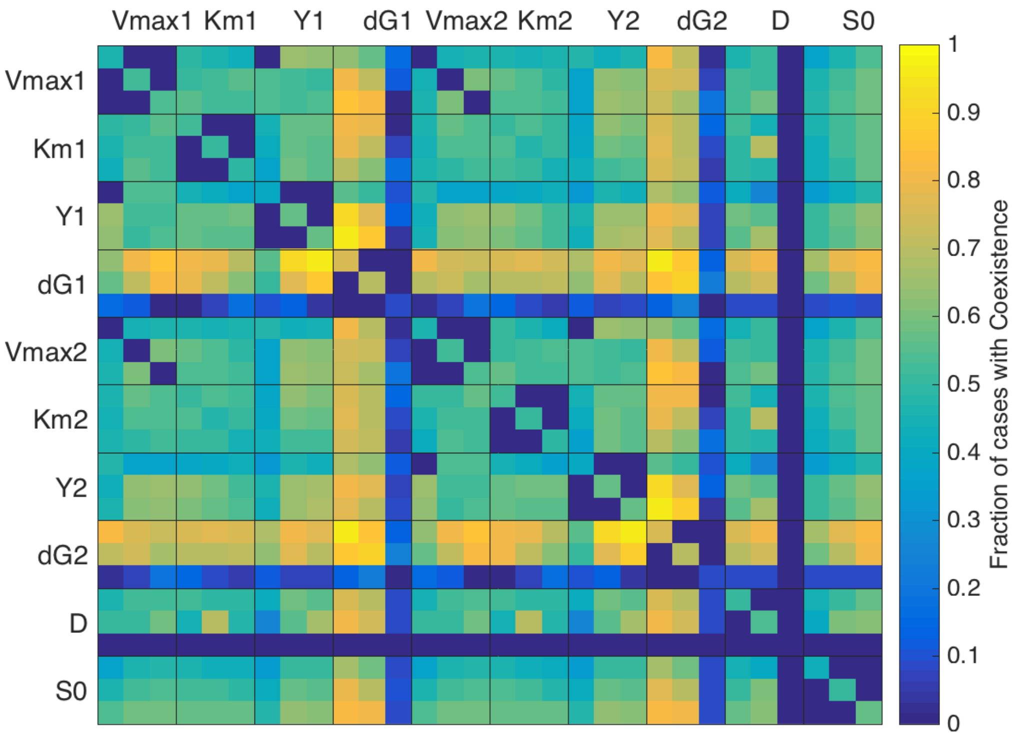

Figure S4. Analysis of steady state species concentrations resulting from the thermodynamic model (*Eq. S2, Eq. S5*) when considering all possible combinations of three different values (low, medium, high) for each of the parameters V_{max1}, K_1, Y_1, dG_1, V_{max2}, K_2, Y_2, dG_2, $S0$ and λ .Each small square in the figure shows the fraction of cases that result in co-existence at steady state (i.e. two species present at steady state with >= 1 µgDW/L) of all feasible solutions for the two-dimensional parameter combination for that square. Parameter values within a 3 x 3 square increase from left to right and from top to bottom. Utilized values are: $V_{max,1}$, $V_{max,2}$: [0.01,0.1,1] (mol/(gDW * h)); K_1, K_2: [1e-6,1e-5,1e-4] (mol/L); Y_1, Y_2: [1,10,100] (gDW/mol); dG_1, dG_2: [-1,-10,-100] (KJ/mol); D (is called λ in the equations): [0.01,0.1,1] (1/h) and S_0: [0.001,0.01,0.1] (mol/L)

References

Boudart M. (1976). Consistency between kinetics and thermodynamics. *J Phys Chem* **80**: 2869–2870.

Curtis GP. (2003). Comparison of approaches for simulating reactive solute transport involving organic degradation reactions by multiple terminal electron acceptors.

Comput Geosci **29**: 319–329.

Heijnen JJ, Van Dijken JP. (1992). In search of a thermodynamic description of biomass yields for the chemotrophic growth of microorganisms. *Biotechnol Bioeng* **39**: 833–58.

Hoh CY, Cord-Ruwisch R. (1996). A practical kinetic model that considers endproduct inhibition in anaerobic digestion processes by including the equilibrium constant. *Biotechnol Bioeng* **51**: 597–604.

Hsu SB, Hubbell S, Waltman P. (1977). A Mathematical Theory for Single-Nutrient Competition in Continuous Cultures of Micro-Organisms. *SIAM J Appl Math* **32**: 366–383.

Jin Q, Bethke CM. (2003). A New Rate Law Describing Microbial Respiration. *Appl Environ Microbiol* **69**: 2340–2348.

Jin Q, Bethke CM. (2007). The thermodynamics and kinetics of microbial metabolism. *Am J Sci* **307**: 643–677.

Kleerebezem R, Stams AJM. (2000). Kinetics of syntrophic cultures: A theoretical treatise on butyrate fermentation. *Biotechnol Bioeng* **67**: 529–543.

Liu C, Zachara JM, Fredrickson JK, Brinkman C, Brinkman CK. (2001). Kinetic Analysis of the Bacterial Reduction of Goethite Kinetic Analysis of the Bacterial Reduction of Goethite. *Environ Sci Technol* **35**: 2482–2490.

Monod J. (1949). The Growth of Bacterial Cultures. *Annu Rev Microbiol* **3**: 371–394.

Rodríguez J, Lema JM, Kleerebezem R. (2008). Energy-based models for environmental biotechnology. *Trends Biotechnol* **26**: 366–374.
