# Supplementary material for: Microbial diversity arising from thermodynamic constraints
Source: ISME J. 2016 Apr 1;10(11):2725–33. doi: 10.1038/ismej.2016.49 (PMC5042319; doi:10.1038/ismej.2016.49)
Supplement: Supplementary Table S1 [file ismej201649x2.docx]

**Table S1**. Mass-Charge balance matrix for the 13 used chemical components.

| **Name** | **Carbon** | **Hydrogen** | **Oxygen** | **Charge** | **dG°_f_** | **Lower bound** | **Upper Bound** |
| --- | --- | --- | --- | --- | --- | --- | --- |
| Glucose | 6 | 12 | 6 | 0 | -917.2 | -1 | -1 |
| Lactate | 3 | 5 | 3 | -1 | -517.8 | 0 | 2 |
| Acetate | 2 | 3 | 2 | -1 | -369.4 | 0 | 3 |
| Carbondioxide | 1 | 0 | 2 | 0 | -394.4 | -6 | 6 |
| Formate | 1 | 1 | 2 | -1 | -351 | 0 | 3 |
| Pyruvate | 3 | 3 | 3 | -1 | -474.6 | 0 | 2 |
| Acetaldehyde | 2 | 4 | 1 | 0 | -139.9 | 0 | 2 |
| Ethanol | 2 | 6 | 1 | 0 | -181.8 | 0 | 2 |
| Hydrogen | 0 | 2 | 0 | 0 | 0 | 0 | 6 |
| Proton | 0 | 1 | 0 | 1 | -40 | -12 | 12 |
| Water | 0 | 2 | 1 | 0 | -237.2 | -6 | 6 |
| Butyrate | 4 | 7 | 2 | -1 | -352.6 | 0 | 2 |
| Methane | 1 | 4 | 0 | 0 | -50.8 | 0 | 6 |
