# Supplementary material for: Microbial diversity arising from thermodynamic constraints
Source: ISME J. 2016 Apr 1;10(11):2725–33. doi: 10.1038/ismej.2016.49 (PMC5042319; doi:10.1038/ismej.2016.49)
Supplement: Supplementary Table S2 [file ismej201649x3.docx]

**Table S2**: List of all reactions used to create Figure 2 with corresponding *ΔG°'* values (1 M concentrations, all gases 1 atm, pH 7, 25°C).

1. 1 Glucose -> 3 Carbondioxide + 3 Methane | dG0 = -418.4

2. 1 Glucose -> 1 Acetate + 2 Carbondioxide + 1 Proton + 2 Methane | dG0 = -382.6

3. 1 Glucose -> 2 Acetate + 1 Carbondioxide + 2 Proton + 1 Methane | dG0 = -346.8

4. 1 Glucose -> 3 Acetate + 3 Proton | dG0 = -311.0

5. 1 Glucose + 2 Water -> 4 Carbondioxide + 4 Hydrogen + 2 Methane | dG0 = -287.6

6. 1 Glucose + 2 Water -> 3 Carbondioxide + 1 Formate + 3 Hydrogen + 1 Proton + 2 Methane | dG0 = -284.2

7. 1 Glucose + 2 Water -> 2 Carbondioxide + 2 Formate + 2 Hydrogen + 2 Proton + 2 Methane | dG0 = -280.8

8. 1 Glucose + 2 Water -> 1 Carbondioxide + 3 Formate + 1 Hydrogen + 3 Proton + 2 Methane | dG0 = -277.4

9. 1 Glucose -> 2 Carbondioxide + 2 Hydrogen + 1 Proton + 1 Butyrate | dG0 = -264.2

10. 1 Glucose + 1 Water -> 3 Carbondioxide + 1 Ethanol + 2 Hydrogen + 1 Methane | dG0 = -261.4

11. 1 Glucose -> 1 Carbondioxide + 1 Formate + 1 Hydrogen + 2 Proton + 1 Butyrate | dG0 = -260.8

12. 1 Glucose + 1 Water -> 2 Carbondioxide + 1 Formate + 1 Ethanol + 1 Hydrogen + 1 Proton + 1 Methane | dG0 = -258.0

13. 1 Glucose -> 2 Formate + 3 Proton + 1 Butyrate | dG0 = -257.4

14. 1 Glucose + 1 Water -> 1 Carbondioxide + 2 Formate + 1 Ethanol + 2 Proton + 1 Methane | dG0 = -254.6

15. 1 Glucose + 2 Water -> 1 Acetate + 3 Carbondioxide + 4 Hydrogen + 1 Proton + 1 Methane | dG0 = -251.8

16. 1 Glucose + 2 Water -> 1 Acetate + 2 Carbondioxide + 1 Formate + 3 Hydrogen + 2 Proton + 1 Methane | dG0 = -248.4

17. 1 Glucose + 2 Water -> 1 Acetate + 1 Carbondioxide + 2 Formate + 2 Hydrogen + 3 Proton + 1 Methane | dG0 = -245.0

18. 1 Glucose + 1 Water -> 1 Lactate + 2 Carbondioxide + 2 Hydrogen + 1 Proton + 1 Methane | dG0 = -243.0

19. 1 Glucose + 2 Water -> 1 Acetate + 3 Formate + 1 Hydrogen + 4 Proton + 1 Methane | dG0 = -241.6

20. 1 Glucose + 1 Water -> 1 Lactate + 1 Carbondioxide + 1 Formate + 1 Hydrogen + 2 Proton + 1 Methane | dG0 = -239.6

21. 1 Glucose + 1 Water -> 1 Lactate + 2 Formate + 3 Proton + 1 Methane | dG0 = -236.2

22. 1 Glucose -> 2 Carbondioxide + 2 Ethanol | dG0 = -235.2

23. 1 Glucose + 1 Water -> 1 Acetate + 2 Carbondioxide + 1 Ethanol + 2 Hydrogen + 1 Proton | dG0 = -225.6

24. 1 Glucose + 1 Water -> 1 Acetate + 1 Carbondioxide + 1 Formate + 1 Ethanol + 1 Hydrogen + 2 Proton | dG0 = -222.2

25. 1 Glucose + 1 Water -> 3 Carbondioxide + 1 Acetaldehyde + 3 Hydrogen + 1 Methane | dG0 = -219.5

26. 1 Glucose + 1 Water -> 1 Acetate + 2 Formate + 1 Ethanol + 3 Proton | dG0 = -218.8

27. 1 Glucose -> 1 Lactate + 1 Carbondioxide + 1 Ethanol + 1 Proton | dG0 = -216.8

28. 1 Glucose + 1 Water -> 2 Carbondioxide + 1 Formate + 1 Acetaldehyde + 2 Hydrogen + 1 Proton + 1 Methane | dG0 = -216.1

29. 1 Glucose + 2 Water -> 2 Acetate + 2 Carbondioxide + 4 Hydrogen + 2 Proton | dG0 = -216.0

30. 1 Glucose + 1 Water -> 1 Carbondioxide + 2 Formate + 1 Acetaldehyde + 1 Hydrogen + 2 Proton + 1 Methane | dG0 = -212.7

31. 1 Glucose + 2 Water -> 2 Acetate + 1 Carbondioxide + 1 Formate + 3 Hydrogen + 3 Proton | dG0 = -212.6

32. 1 Glucose + 1 Water -> 3 Formate + 1 Acetaldehyde + 3 Proton + 1 Methane | dG0 = -209.3

33. 1 Glucose + 2 Water -> 2 Acetate + 2 Formate + 2 Hydrogen + 4 Proton | dG0 = -209.2

34. 1 Glucose + 1 Water -> 1 Lactate + 1 Acetate + 1 Carbondioxide + 2 Hydrogen + 2 Proton | dG0 = -207.2

35. 1 Glucose + 1 Carbondioxide + 2 Water -> 2 Acetate + 3 Formate + 1 Hydrogen + 5 Proton | dG0 = -205.8

36. 1 Glucose + 1 Water -> 1 Lactate + 1 Acetate + 1 Formate + 1 Hydrogen + 3 Proton | dG0 = -203.8

37. 1 Glucose + 1 Carbondioxide + 1 Water -> 1 Lactate + 1 Acetate + 2 Formate + 4 Proton | dG0 = -200.4

38. 1 Glucose + 1 Water -> 2 Carbondioxide + 1 Pyruvate + 3 Hydrogen + 1 Proton + 1 Methane | dG0 = -199.8

39. 1 Glucose -> 2 Lactate + 2 Proton | dG0 = -198.4

40. 1 Glucose + 1 Water -> 1 Carbondioxide + 1 Formate + 1 Pyruvate + 2 Hydrogen + 2 Proton + 1 Methane | dG0 = -196.4

41. 1 Glucose -> 2 Carbondioxide + 1 Acetaldehyde + 1 Ethanol + 1 Hydrogen | dG0 = -193.3

42. 1 Glucose + 1 Water -> 2 Formate + 1 Pyruvate + 1 Hydrogen + 3 Proton + 1 Methane | dG0 = -193.0

43. 1 Glucose -> 1 Carbondioxide + 1 Formate + 1 Acetaldehyde + 1 Ethanol + 1 Proton | dG0 = -189.9

44. 1 Glucose + 1 Carbondioxide + 1 Water -> 3 Formate + 1 Pyruvate + 4 Proton + 1 Methane | dG0 = -189.6

45. 1 Glucose + 1 Water -> 1 Acetate + 2 Carbondioxide + 1 Acetaldehyde + 3 Hydrogen + 1 Proton | dG0 = -183.7

46. 1 Glucose + 1 Water -> 1 Acetate + 1 Carbondioxide + 1 Formate + 1 Acetaldehyde + 2 Hydrogen + 2 Proton | dG0 = -180.3

47. 1 Glucose + 1 Water -> 1 Acetate + 2 Formate + 1 Acetaldehyde + 1 Hydrogen + 3 Proton | dG0 = -176.9

48. 1 Glucose -> 1 Lactate + 1 Carbondioxide + 1 Acetaldehyde + 1 Hydrogen + 1 Proton | dG0 = -174.9

49. 1 Glucose -> 1 Carbondioxide + 1 Pyruvate + 1 Ethanol + 1 Hydrogen + 1 Proton | dG0 = -173.6

50. 1 Glucose + 1 Carbondioxide + 1 Water -> 1 Acetate + 3 Formate + 1 Acetaldehyde + 4 Proton | dG0 = -173.5

51. 1 Glucose -> 1 Lactate + 1 Formate + 1 Acetaldehyde + 2 Proton | dG0 = -171.5

52. 1 Glucose -> 1 Formate + 1 Pyruvate + 1 Ethanol + 2 Proton | dG0 = -170.2

53. 1 Glucose + 1 Water -> 1 Acetate + 1 Carbondioxide + 1 Pyruvate + 3 Hydrogen + 2 Proton | dG0 = -164.0

54. 1 Glucose + 1 Water -> 1 Acetate + 1 Formate + 1 Pyruvate + 2 Hydrogen + 3 Proton | dG0 = -160.6

55. 1 Glucose + 1 Carbondioxide + 1 Water -> 1 Acetate + 2 Formate + 1 Pyruvate + 1 Hydrogen + 4 Proton | dG0 = -157.2

56. 1 Glucose -> 1 Lactate + 1 Pyruvate + 1 Hydrogen + 2 Proton | dG0 = -155.2

57. 1 Glucose + 2 Carbondioxide + 1 Water -> 1 Acetate + 3 Formate + 1 Pyruvate + 5 Proton | dG0 = -153.8

58. 1 Glucose + 1 Carbondioxide -> 1 Lactate + 1 Formate + 1 Pyruvate + 3 Proton | dG0 = -151.8

59. 1 Glucose -> 2 Carbondioxide + 2 Acetaldehyde + 2 Hydrogen | dG0 = -151.4

60. 1 Glucose + 4 Water -> 3 Carbondioxide + 2 Formate + 6 Hydrogen + 2 Proton + 1 Methane | dG0 = -150.0

61. 1 Glucose -> 1 Carbondioxide + 1 Formate + 2 Acetaldehyde + 1 Hydrogen + 1 Proton | dG0 = -148.0

62. 1 Glucose + 4 Water -> 2 Carbondioxide + 3 Formate + 5 Hydrogen + 3 Proton + 1 Methane | dG0 = -146.6

63. 1 Glucose -> 2 Formate + 2 Acetaldehyde + 2 Proton | dG0 = -144.6

64. 1 Glucose -> 1 Carbondioxide + 1 Pyruvate + 1 Acetaldehyde + 2 Hydrogen + 1 Proton | dG0 = -131.7

65. 1 Glucose + 3 Water -> 4 Carbondioxide + 1 Ethanol + 6 Hydrogen | dG0 = -130.6

66. 1 Glucose -> 1 Formate + 1 Pyruvate + 1 Acetaldehyde + 1 Hydrogen + 2 Proton | dG0 = -128.3

67. 1 Glucose + 3 Water -> 3 Carbondioxide + 1 Formate + 1 Ethanol + 5 Hydrogen + 1 Proton | dG0 = -127.2

68. 1 Glucose + 1 Carbondioxide -> 2 Formate + 1 Pyruvate + 1 Acetaldehyde + 3 Proton | dG0 = -124.9

69. 1 Glucose + 3 Water -> 2 Carbondioxide + 2 Formate + 1 Ethanol + 4 Hydrogen + 2 Proton | dG0 = -123.8

70. 1 Glucose + 3 Water -> 1 Carbondioxide + 3 Formate + 1 Ethanol + 3 Hydrogen + 3 Proton | dG0 = -120.4

71. 1 Glucose + 4 Water -> 1 Acetate + 2 Carbondioxide + 2 Formate + 6 Hydrogen + 3 Proton | dG0 = -114.2

72. 1 Glucose + 3 Water -> 1 Lactate + 3 Carbondioxide + 6 Hydrogen + 1 Proton | dG0 = -112.2

73. 1 Glucose -> 2 Pyruvate + 2 Hydrogen + 2 Proton | dG0 = -112.0

74. 1 Glucose + 4 Water -> 1 Acetate + 1 Carbondioxide + 3 Formate + 5 Hydrogen + 4 Proton | dG0 = -110.8

75. 1 Glucose + 3 Water -> 1 Lactate + 2 Carbondioxide + 1 Formate + 5 Hydrogen + 2 Proton | dG0 = -108.8

76. 1 Glucose + 1 Carbondioxide -> 1 Formate + 2 Pyruvate + 1 Hydrogen + 3 Proton | dG0 = -108.6

77. 1 Glucose + 3 Water -> 1 Lactate + 1 Carbondioxide + 2 Formate + 4 Hydrogen + 3 Proton | dG0 = -105.4

78. 1 Glucose + 2 Carbondioxide -> 2 Formate + 2 Pyruvate + 4 Proton | dG0 = -105.2

79. 1 Glucose + 3 Water -> 1 Lactate + 3 Formate + 3 Hydrogen + 4 Proton | dG0 = -102.0

80. 1 Glucose + 3 Water -> 3 Carbondioxide + 1 Formate + 1 Acetaldehyde + 6 Hydrogen + 1 Proton | dG0 = -85.3

81. 1 Glucose + 3 Water -> 2 Carbondioxide + 2 Formate + 1 Acetaldehyde + 5 Hydrogen + 2 Proton | dG0 = -81.9

82. 1 Glucose + 3 Water -> 1 Carbondioxide + 3 Formate + 1 Acetaldehyde + 4 Hydrogen + 3 Proton | dG0 = -78.5

83. 1 Glucose + 3 Water -> 2 Carbondioxide + 1 Formate + 1 Pyruvate + 6 Hydrogen + 2 Proton | dG0 = -65.6

84. 1 Glucose + 3 Water -> 1 Carbondioxide + 2 Formate + 1 Pyruvate + 5 Hydrogen + 3 Proton | dG0 = -62.2

85. 1 Glucose + 3 Water -> 3 Formate + 1 Pyruvate + 4 Hydrogen + 4 Proton | dG0 = -58.8
